# Role of Self-Efficacy and Resistance to Innovation on the Demotivation and Insufficient Learning Capabilities of Preservice English Normal Students in China

**DOI:** 10.3389/fpsyg.2022.923466

**Published:** 2022-07-29

**Authors:** Tuanhua Lu, Mohd Yusof Sanitah, Yongliang Huang

**Affiliations:** ^1^School of Foreign Languages, Xianyang Normal University, Xianyang, China; ^2^School of Education, Faculty of Social Science and Humanities, Universiti Teknologi Malaysia, Johor Bahru, Malaysia

**Keywords:** self-efficacy, resistance to innovation, demotivation, insufficient learning capabilities, preservice English normal students

## Abstract

Learning capabilities among students are the crucial element for the student’s success in learning a particular language, and this phenomenon needs recent studies. The current study examines the impact of self-efficacy and resistance to innovation on the demotivation and insufficient learning capabilities of preservice English normal students in China. The current research also investigates the mediating impact of demotivation among self-efficacy, resistance to innovation, and insufficient learning capabilities. The questionnaires were employed by the researchers to gather the data from chosen respondents. The preservice English students are the respondents of the study. These are selected using purposive sampling. These questionnaires were forwarded to them by personal visits. The researchers have sent 690 surveys but only received 360 surveys and used them for analysis. These surveys represented a 52.17% response rate. The SPSS-AMOS was applied to test the relationships among variables and also test the hypotheses of the study. The results revealed that self-efficacy and resistance to innovation have a significant and a positive linkage with demotivation and insufficient learning capabilities. The findings also indicated that demotivation significantly mediates self-efficacy, resistance to innovation, and insufficient learning capabilities. The article helps the policymakers to establish the regulations related to the improvement of learning capabilities using innovation adoption and motivation of the students.

## Introduction

China has the greatest number of English learners (Rose et al., 2020). Recently, the intervention of the English language in the Chinese education system accelerated at a rapid pace (Cheng and Chen, 2021). One of the reasons is the increasing interest of the world in China due to different reasons like education, business, jobs, etc. English has become a compulsory part of the Chinese curriculum for all levels like elementary, middle, high school, colleges, and universities. The preschool in China is adding English to their curriculum. The time the Chinese students graduate from the universities they have at least studied English for about 2,000 h over the duration of 10 years. The requirement to study English is defined in a true manner in an article titled Crazy English: Good or Bad: “Whether [English] is helpful or not, you must learn it; whether you learn it or not, you will never be able to utilize it.” In China, it appears that learning English has hit a snag. Despite putting in a lot of effort to study the language, English learners have not gained the ability to use it. One key factor for Chinese learners’ lack of communicative competency has been identified in the school’s teaching method. In China, English language instruction is frequently oriented on the instructor, the textbook, and grammar. In other words, English instruction in China is primarily concerned with mastery of grammar and vocabulary as taught by the teacher through the use of a textbook. This form of teaching strategy fails the generation of appropriate teaching skills in students. In this context: the Chinese government has attempted to strengthen English education in the school system in the 21st century.

The literature strongly proposed that educational institution plays a vital role in the betterment of English language learning in China (Liu et al., 2021). The Chinese education sector introduced the New English Curriculum (NEC) for elementary and secondary schools in 2001. The NEC argues for pedagogical reform to address the dilemma in which teachers stress linguistic knowledge while ignoring students’ actual proficiency in using English. It states that when teaching, English teachers should take into account their students’ interests, experiences, and cognition. To improve English learners’ synthesis skills in using the language, the NEC suggests a variety of instructional approaches, such as participation, cooperation, and discussion. In a nutshell, the NEC encourages communicative language teaching. Teachers play an important part in this pedagogy change because they determine if curricular innovations can be successfully implemented in the classroom as desired by legislators (Chien et al., 2021a). Teachers are the key to the deliverance of the right knowledge at the right time in the right way to the right people. The teacher’s method of teaching results in students’ motivation of demotivation. The educational institution leads to implement the new way of teaching to meet the world. The teachers make it enable to not only accept but also educate the students to absorb this change. Many times this change in terms of innovation leads to students’ demotivation which directly affects the learning of a second language (Gao and Ren, 2019; Chien et al., 2021b; Liu et al., 2021). This is one of the reasons to check the reason for insufficient learning capabilities in Chinese second language learning schools.

The present study will address some gaps does exist in the literature like (1) being one of the important topics like linguistic along with learning capabilities although researched although but still not reached its peak, (2) Wu et al. (2021) worked on the students learning capabilities development whereas the present study will work on insufficient learning capabilities along with mediation effect of demotivation in China, (3) Jamil et al. (2021) checked the computer interference in enhancement of students capabilities whereas the present study will work on insufficient learning capabilities along with self-efficacy, resistance to innovation, and demotivation in China, (4) Amarakoon et al. (2018) worked on the learning capabilities along with innovation whereas the present study will test the insufficient learning capabilities along with number of other variables in Chinese perspective with a new data set, (5) the present study will check the model in Chinese perspective with new data set, and (6) Bonal and González (2020) worked on the lockdown and learning whereas the present study will investigate the insufficient learning capabilities with mediation effect of demotivation in China. The contributions of the study are (1) highlight the importance of preservice English learning capabilities in China, (2) help professionals revamp their policies for the betterment of the learning capabilities of preservice individuals, (3) help the researchers to identify and explore the more aspects of the lack of learning capabilities, (4) it provides the help to the policymakers in developing the policies related to the learning capabilities, and (5) it facilities the relevant authorities to implement the effective policies to improve the learning capabilities.

The study structure is divided into five phases. The first phase will present the introduction. In the second phase of the study, the pieces of evidence regarding low self-efficacy, low self-esteem, resistance to innovation, and self-demotivation will be discussed in the light of past literature. The third phase of the study will shine the spotlight on the methodology employed for the collection of data regarding low self-efficacy, low self-esteem, resistance to innovation, and self-demotivation and its validity will be analyzed. In the fourth phase, the results of the study will be compared with the pieces of evidence reviewed from the literature. In the last phase, the study implications along with the conclusion and future recommendations will be presented which will conclude the article.

## Literature Review

Self-efficacy, proposed by Albert Bandura, refers to an individual’s belief in their capability to perform behaviors essential to produce definite performance achievements (Gielnik et al., 2020). In addition, demotivation refers to the lack of one’s interest, enthusiasm, and willingness to perform an action due to specific negative influences (Albalawi and Al-Hoorie, 2021). Moreover, the learning capabilities and innovation encompass skills, knowledge, skills, and dispositions. Learners develop capability when they apply skills and knowledge effectively in changing circumstances in their learning (Lee and Falahat, 2019).

The performance of learning capabilities and academic performance are dependent on the important factors of self-efficacy. Learning capabilities among students are the crucial element for the student’s success in learning a particular language, and this phenomenon needs recent studies. For the improvement of learning capabilities, it is most important for educational institutions to promote the factors of self-efficacy. Gundel et al. (2019) elaborated on the simulations of mixed reality in self-efficacy for the preservice of education of teachers for learning capabilities. Self-efficacy leads to the individual that entails higher beliefs in their performance and the commitments in China. Any sort of lower self-efficacy leads to failures in lack of attempts and the lack of abilities. Ulenski et al. (2019) assessed the validating and developing literacy that coaches the survey of self-efficacy influencing the learning capabilities of students. Through effective perseverance, endeavors, and commitments, self-efficacy leads to the excellent performance of individuals. The students that entail higher self-efficacy are significant and positive in influencing the insufficient learning capabilities. For the fulfillment of academic tasks and educational performance, the factors related to self-efficacy must be promoted in the educational sectors of China. van Rooij et al. (2019) enumerated the sources of teachers’ self-efficacy with the teaching careers influencing the demotivation. Among the students of preservice English learning, the role of self-efficacy determines higher values for the elimination of demotivation. To overcome the challenges and attain higher values and objectives, the students are required to acquaint themselves with self-efficacy. Harrison et al. (2018) investigated the capabilities, participation, and access of self-efficacy that are important for the flourishment of learning capabilities. Academic self-efficacy involves a greater role and has a significant and positive impact on the demotivation of students. The probable and effective entailment of self-efficacy in the students leads to effective student learning, academic performance, and motivation. The considerable involvement and inducement of self-efficacy is especially the integrated and dominant mean for the students which influences the demotivation. Afshari and Hadian Nasab (2021) examined the learning capabilities by the managing talent of intellectual capital and self-efficacy that enhances the organizational environment. The effective mechanisms and modes endorse motivational and cognitive approaches to the self-efficacy that speak emotions of Chinese students in their achievements and motivation. Learning strategies must be organized in such a way that self-efficacy must be uplifted. The uplifting of self-efficacy facilitates the students and individual learning of normal students to eradicate the insufficient learning capabilities. Thus, the below-given hypothesis is derived from the above debate.

**H1:** Self-efficacy significantly influences insufficient learning capabilities.

**H2:** Self-efficacy significantly influences demotivation.

Countries that are developing and developed have certain barriers to innovation and insufficient learning (Chien et al., 2021c). This is due to the lack of facilities and lack of technological introduction in educational institutions. There are many barriers to innovation that lead to insufficient learning capabilities. Park et al. (2018) narrated the resistance, perceived attributes, and system quality necessary in education to provide sufficient learning capabilities. Mostly, the busy parents and lack of money is also considered the main element that leads to the resistance to innovation. This also states the involvement of backward areas of China which are not fond of technology and are not acquainted with its use of it. Caiazza and Volpe (2017) discussed the actions, actors, and process of diffusion of resistance narratives and uplifting the innovation for learning capabilities. Therefore, the resistance to innovation leads to many other circumstances which are negative for the learning capabilities. In the preservice English normal students, the resistance to innovation is encouraging a lack of education and a lack of curriculum of innovation. This depicts the image of disadvantage to the innovation which is a better model for the English learners of China. Pedler and Brook (2017) analyzed the innovation and action learning that requires implementation, engagement, and elimination of resistance. As the English language is considered a global language all over the world and lack of facilities could lead to insufficient learning. The lack of insufficient learning not only distracts the abilities of students but also depicts the damaging circumstances toward the learning capabilities. Dejaeghere (2020) investigated the concept and capabilities that are related to the equalities and inequalities due to the resistance to innovation. Students are inspired by the examples and technology that is the better tool for them to attain sufficient learning. Sufficient learning not only enhances the capabilities of students but also poses a dominant image of the resistance to innovation in China. Presbitero et al. (2017) narrated various dynamics of organizational learning capabilities by the knowledge sharing capabilities after removing barriers to innovation. The resistance to innovation is considered a curriculum barrier to the innovation which eliminates the creative environments for enhancing the learning capabilities. The resistance to innovation majorly dominates the educational learning and preservice English students could not attain sufficient learning.

The perceptions of personal efficacy refer to the individual capabilities and self-efficacy which entails the involvement of demotivation. Demotivation is promotive in the educational sector of China that states the elements in students learning which can be destructive. Demotivation among the students leads to a lack of self-efficacy that enumerates the capabilities related to insufficient learning. Pathan et al. (2021) investigated the demotivation factors associated with language learning among university students due to the resilience and matter of personality. The factors associated with demotivation entail mediating impact on the preservice students learning. Many normal students in the schools, colleges, and universities when demotivated by the lack of educational facilities depicts the insufficient learning capabilities. It is important for the institutions to develop certain capabilities which are deemed requirements to enhance English learning in China. Kiel et al. (2020) enumerated the inclusiveness of classes by the teachers and students’ self-efficacy and its role toward learning capabilities with institutional support. Various preservice English normal students are required to develop the capabilities of motivation that also increases the element of self-efficacy among them. Whitfield and Staritz (2021) discussed various traps of learning that arise due to the factors of demotivation and insufficient materials for learning. The increasing trend of demotivation has a dominant impact on the relationship between insufficient learning capabilities and self-efficacy. When there is a lack of self-efficacy there could be insufficient learning capabilities. It is only due to the demotivation which is increased in the students due to unfortunate learning capabilities and abilities of the teachers. Usually, the students are when demotivated by the learning facilities and the distraction among them could be negative to their upbringing. Ghanizadeh and Jahedizadeh (2017) examined the relationship between motivational facts and metacognitive and emotional facts that arise after the demotivation in university students. Not only in the educational sectors but also in the communities of China, creative approaches and innovation must be promoted. The promotion of innovation and creativity enables the communities in discovering new markets and strategies. While the students could also be enabled with taking up new ideas and development of skills and knowledge, especially in the English. Thus, the below-given hypothesis is derived from the above debate.

**H3:** Resistance to innovation significantly influences insufficient learning capabilities.

**H4:** Resistance to innovation significantly influences demotivation.

The establishment of different platforms and programs for English learners is encouraged by the effective use of innovative techniques. The tools and technology that are resisted while providing English learning to the preservice students also denominate the insufficient learning capabilities. Rizvi and Nabi (2021) emphasized the transformation of learning capabilities by the existence of challenges and issues that states the prevalence of demotivation. Among the students of English, the inspiration of technology and the motivation to deal with risk and ideas not only enhances their capabilities but also raises their independency. It is necessary for the educational sectors to induce certain changes and encourage motivation to enable the elements toward resistance to innovation. This resistance to innovation somehow leads to insufficient learning capabilities and the demotivation extended the central role among them. Dakka (2020) investigated the diversity, innovation, and competition in higher education where the resistance dominates with higher values with the involvement of demotivation. Some creative approaches to the development of technology and innovation are important in uplifting learning capabilities. The preservice English normal students in China are when demotivated the students then have insufficient learning capabilities. Self-determination is promotive and positive for the endorsement and raising of independency and determination in preservice English normal students. Wilson-Strydom (2017) assessed the inequalities and equalities with the capabilities and resilience to innovation that could be disrupting the learning capabilities with the involvement of demotivation. Students are required to be well-acquainted with the creative curriculum innovation and technology. This technological advancement, however, increases the elimination of factors that are associated with demotivation and insufficient learning capabilities. The innovative learning environment for the preservice students of English is enhanced with the wider support of motivation. Inan and Karaca (2021) narrated different practices of quality assurance in the learning capabilities of young learners due to the presence of demotivation. The learning capabilities could attain significant enhancement when the students are self-determined and motivated by their teachers and educational facilities in China. Mostly the learning abilities and capabilities are dependent on digital innovation because feasible innovation provides more education in a short time. The certain and fortunate mediating impact of demotivation is bringing a narrative of centered role in self-efficacy and insufficient learning capabilities. Thus, the below-given hypothesis is derived from the above debate.

**H5:** Demotivation significantly and positively mediates the relationship between self-efficacy and insufficient learning capabilities.

**H6:** Demotivation significantly and positively mediates the relationship between resistance to innovation and insufficient learning capabilities.

## Research Methodology

**FIGURE 1:**
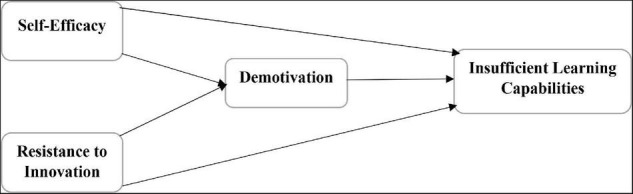
Theoretical model.

The study examines the impact of self-efficacy and resistance to innovation on the demotivation and insufficient learning capabilities and also investigates the mediating impact of demotivation among self-efficacy, resistance to innovation, and insufficient learning capabilities of preservice English normal students in China. The current article has the motive of predicting the variables and adopted the deductive approach. In addition, the study motives also include the quantitative examination of the respondents and used the quantitative method like surveys to collect the data from respondents. The questionnaires were employed by the researchers to gather the data from chosen respondents. The present article has used two independent constructs, such as self-efficacy and resistance to innovation. In addition, the present article has also used demotivation as the mediating variable, and insufficient learning capabilities have been used as the dependent variable. These variables are presented in the theoretical framework in Figure 1.

**FIGURE 2:**
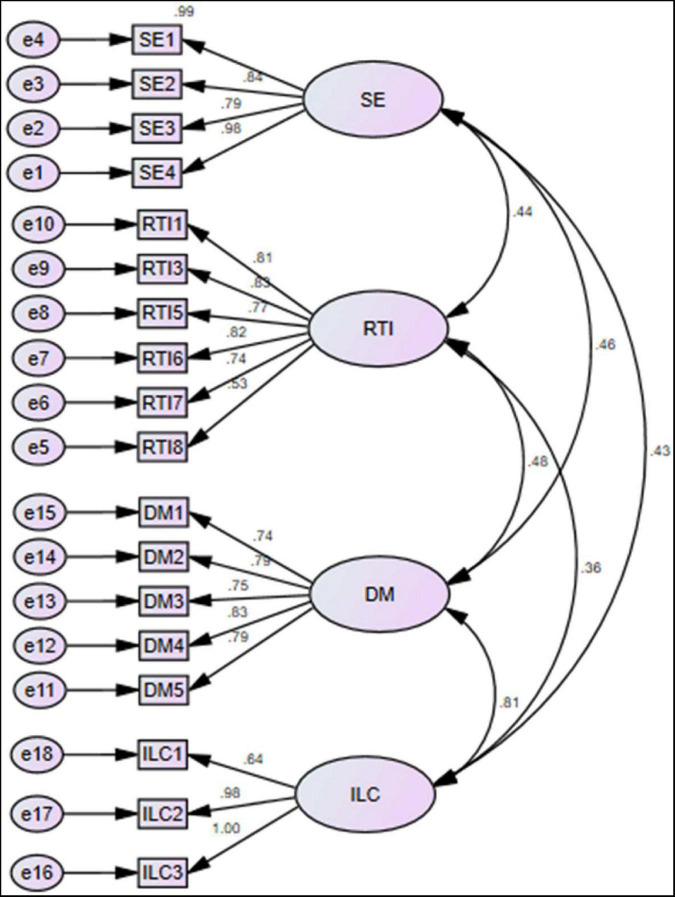
Measurement model assessment. Indicated that the factor loadings of the items are larger than 0.50 and indicated valid convergent validity.

**FIGURE 3:**
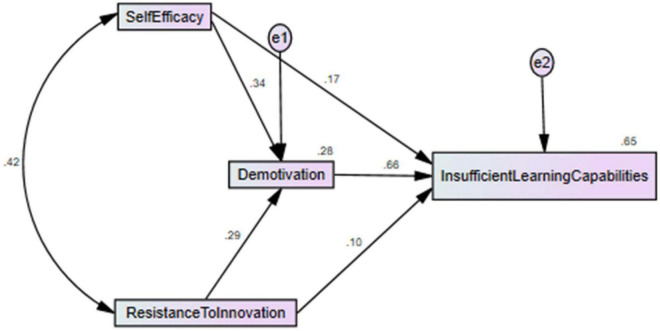
Structural model assessment.

The questionnaires were taken from the past studies, like self-efficacy is the independent variable that has four items taken from Yavuzalp and Bahcivan (2020). In addition, resistance to innovation has also been taken as a predictor that has eight items extracted from Mani and Chouk (2018). Moreover, the current article has used demotivation as the mediating variable with five items taken from Pillny et al. (2018). Finally, insufficient learning capabilities have been used as a dependent variable that has three items taken from Gieske et al. (2019). In addition, the preservice English students are the respondents of the study. These are selected using purposive sampling. The study selected only those students who are near to joining the professional life. Figure 2 show the content validity and the figures indicated that the factor loading values are larger than 0.50 and exposed valid content validity. These questionnaires were forwarded to them by personal visits. The researchers have sent 690 surveys but only received 360 surveys and used them for analysis. These surveys represented a 52.17% response rate. Moreover, the SPSS-AMOS was applied to test the relationships among variables and also test the hypotheses of the study. It is the best estimation tool that provides the best findings even the large sample sizes used by the authors or complex model has been selected by the researchers (Hair et al., 2020). The measurement model exposed the construct validity and reliability that is checked using average variance extracted (AVE), composite reliability (CR), and factor loadings. The minimum threshold for AVE is that it should be greater than 0.50 (Nasution et al., 2020), while the minimum threshold for CR is that it should be greater than 0.70 (Kamis et al., 2020), and the minimum threshold for factor loadings is that it should be greater than 0.50 (Dai et al., 2019). Finally, the structural model exposed the associations among the variables. Figure 3 indicated that the self-efficacy and resistance to innovation have positive association with insufficient learning capabilities and demotivation significantly mediates among them.

## Research Findings

The current article has examined the content validity with the help of factor loadings. The results indicated that the values are not less than 0.50. These values reported that the content validity proved as valid. In addition, the current article has examined the convergent validity with the help of AVE. The results indicated that the values are not less than 0.50. These values reported that the convergent validity proved as valid. Moreover, the current article has examined the reliability with the help of CR. The results indicated that the values are not less than 0.70. These values reported that the reliability proved as valid. Table 1 shows the convergent validity results.

**TABLE 1:** Convergent validity.

Constructs	Items	Loadings	CR	AVE
Self-efficacy	SE4	0.983	0.948	0.822
	SE3	0.790		
	SE2	0.842		
	SE1	0.994		
Resistance to innovation	RTI8	0.530	0.888	0.573
	RTI7	0.741		
	RTI6	0.821		
	RTI5	0.771		
	RTI3	0.828		
	RTI1	0.809		
Demotivation	DM5	0.788	0.885	0.606
	DM4	0.827		
	DM3	0.746		
	DM2	0.787		
	DM1	0.740		
Insufficient learning capabilities	ILC3	0.999	0.916	0.790
	ILC2	0.983		
	ILC1	0.637		

The current article has also examined the discriminant validity with the help of Fornell Larcker. The results indicated that the first value in the column is higher than the other values in the same column. These values reported that the stronger nexus with the variable itself and discriminant validity proved as valid. Table 2 shows the discriminant validity results.

**TABLE 2:** Discriminant validity.

	ILC	SE	RTI	DM
ILC	0.889			
SE	0.426	0.907		
RTI	0.358	0.444	0.757	
DM	0.809	0.461	0.480	0.673

The study examines the impact of self-efficacy and resistance to innovation on the demotivation and insufficient learning capabilities and also investigates the mediating impact of demotivation among self-efficacy, resistance to innovation, and insufficient learning capabilities of preservice English normal students in China. The study examines the impact of self-efficacy and resistance to innovation on the demotivation and insufficient learning capabilities and also investigates the mediating impact of demotivation among self-efficacy, resistance to innovation, and insufficient learning capabilities of preservice English normal students in China. The results revealed that self-efficacy and resistance to innovation have a significant and a positive linkage with insufficient learning capabilities and accept H1 and H3. In addition, the results also revealed that self-efficacy and resistance to innovation have a significant and a positive linkage with demotivation and accept H2 and H4. Table 3 shows the direct association among variables results.

**TABLE 3:** A path analysis.

Relationships			Std. beta	Beta	S.E.	C.R.	*P*
Demotivation	←	Resistance to innovation	0.299	0.274	0.048	5.743	***
Demotivation	←	Self-Efficacy	0.358	0.258	0.037	6.882	***
Insufficient learning capabilities	←	Self-Efficacy	0.178	0.123	0.027	4.498	***
Insufficient learning capabilities	←	Resistance to innovation	0.108	0.095	0.034	2.788	0.005
Insufficient learning capabilities	←	Demotivation	0.664	0.639	0.040	16.042	***

***, **, and *represent significant level at 1, 5, and 10%, respectively.

The findings also indicated that demotivation significantly mediates self-efficacy, resistance to innovation, and insufficient learning capabilities and accept H5 and H6. Table 4 shows the indirect association among variables results.

**TABLE 4:** Mediation analysis.

	SE		RTI	
	Beta	*P*-values	Beta	*P*-values
Total effects	0.543	0.000	0.433	0.000
Direct effects	0.622	0.000	0.134	0.002
Indirect effects	0.533	0.000	0.625	0.000

## Discussion

The study examines the impact of self-efficacy and resistance to innovation on the demotivation and insufficient learning capabilities and also investigates the mediating impact of demotivation among self-efficacy, resistance to innovation, and insufficient learning capabilities of preservice English normal students in China. The results indicated that self-efficacy has a significant and a positive association with the insufficient learning capabilities among preservice English normal students in China. The students that are considered self-efficient and have self-efficacy are not competent enough and have insufficient learning capabilities, which is the reason for the positive association between self-efficacy and insufficient learning capabilities. This outcome is in line with Hatlevik and Hatlevik (2018), who also investigated the nexus between self-efficacy and insufficient learning capabilities and revealed that self-efficacy among the students does not always work positively on the learning capabilities. Sometimes students have enough self-efficacy but fail to attain sufficient learning capabilities. In addition, a study by Zamani-Alavijeh et al. (2019) also examined the self-efficacy role on the learning capabilities and revealed that the self-efficacy among students had put a negative impact on the learning capabilities because the self-efficacy among students miss interpret their qualities and fail to gain sufficient learning capabilities and this outcome is similar to the current study findings. Moreover, this output is also same as the Kuyini et al. (2020), who also exposed the self-efficacy impact on the learning capabilities and exposed that the self-efficacy among students makes them selfish and they put lack of effort results in insufficient learning capabilities.

The outcome also exposed that the resistance to innovation has a positive role on the insufficient learning capabilities among preservice English normal students in China. The lack of focus on innovation leads the students to a less attractive environment, which creates insufficient learning capabilities among students. Innovation adoption always brings new ideas that polish the students and enhance their learning capabilities and vice versa. This result is matched with Kim et al. (2018), who also analyzed the innovation impact on the learning capabilities and revealed that the innovation adoption always changes the existing processes and enables the student to gain sufficient learning capabilities and vice versa. In addition, this result is also the same as Shahbaz et al. (2019), who also investigated the resistance to change impact on the learning abilities and exposed that the learning abilities could be enhanced by the adoption of new technology in the existing process, but resistance toward change always restrict the students and face insufficient learning capabilities. In addition, this outcome is in line with Choi and Chandler (2020), who also analyzed the association between resistance to change and learning abilities and indicated that the high learning capabilities could be achieved by adopting innovation in the process, but if the students are reluctant to adopt innovation, then they faced insufficient learning abilities.

The results also indicated that self-efficacy has a significant and a positive association with the demotivation among preservice English normal students in China. The students who are considered self-efficient and have self-efficacy are sometimes demotivated by the existing way of working, which is the reason for the positive association between self-efficacy and demotivation. This outcome is in line with Tannady et al. (2019), who also investigated the nexus between self-efficacy and motivation and revealed that self-efficacy among the student does not always work positively on the motivation. Sometimes students have enough self-efficacy but fail to get motivation. In addition, a study by Rhew et al. (2018) also examined the self-efficacy role in motivation. It revealed that self-efficacy among students has a negative impact on motivation because self-efficacy among students misses interpreting their qualities and fails to gain motivation. This outcome is similar to the current article outcomes. Moreover, the results are in line with Torres and Alieto (2019), who indicated that the self-efficacy among students makes them selfish, and they put lack of effort and demotivated in the existing way of workings.

The outcome also exposed that the resistance to innovation positively affects the demotivation among preservice English normal students in China. The lack of focus on innovation leads the students to a less attractive environment, which creates demotivation among students. Innovation adoption always brings new ideas that polish the students and enhance their motivation and vice versa. This result is matched with Bonta (2019), who also analyzed the innovation impact on the motivation and revealed that the innovation adoption always brings the changes in the existing processes and creates motivation among the students. In addition, this result is also the same as Hashimy et al. (2021), who also investigated the resistance to change impact on the motivation and exposed that the motivation could be enhanced by the adoption of new technology in the existing process, but resistance toward change always restrict the students and face lack of motivation among them. In addition, this outcome is in line with Fischer et al. (2019), who also analyzed the association between innovation and motivation and indicated that the high motivation could be achieved by adopting innovation in the process, but if the students are reluctant to adopt innovation then they are demotivated and fail to achieve the desired goals.

The results also revealed that demotivation significantly and positively mediates self-efficacy and insufficient learning capabilities among preservice English normal students in China. The students who have self-efficacy but are not interested in a particular field create demotivation among them and lead to insufficient learning capabilities. This outcome is in line with Shin (2018), who also examines the self-efficacy and motivation role in learning capabilities and revealed that sometimes self-efficacy does not work to motivate the students in a particular task, leading to insufficient abilities to perform that task. This result is also similar to the Haerazi and Irawan (2020), who also investigated the self-efficacy role in motivation that lead to learning abilities and indicated that the self-efficacy among students sometimes has a negative impact on the motivation and also has a negative influence on the learning capabilities of the students.

The findings also revealed that the demotivation significantly and positively mediates resistance to innovation and insufficient learning capabilities among preservice English normal students in China. This outcome is in line with Oke and Fernandes (2020), who also examined the innovation and motivation role in the learning capabilities and revealed that the resistance to innovation creates demotivation among the students, and this also creates insufficient learning abilities among them. This result is also similar to the Amarakoon et al. (2018), who also investigated the innovation role in motivation that lead to learning abilities and indicated that the adoption of innovation creates motivation among students and that gain sufficient learning capabilities, but if they resist adopting innovation then it creates demotivation among students and put a negative influence on the learning capabilities of the students.

### Conclusion

The study concluded that the students in the education sector of China lack self-efficacy, which is the reason for their demotivation and insufficient learning capabilities among them. In addition, the study also concluded that the students also have the behavior of resistance to innovation in the Chinese education institution and produce insufficient learning capabilities among the students. Moreover, the students are demotivated in the English language learning institutions that produce insufficient learning capabilities among students. Finally, the article also concluded that the lack of self-efficacy and resistance to innovation nature reduce the motivation and enhance the insufficient learning capabilities among students.

### Implications and Limitations

This article has several theoretical contributions and also significant implications. The current article contributes to the literature by conducting an examination of self-efficacy and insufficient learning capabilities. In addition, the article also contributes to the existing literature by providing the investigation of resistance to innovation and self-learning capabilities. Moreover, the demotivation is used as the mediating variable among self-efficacy, demotivation, and insufficient learning capabilities are significant contributions in the existing literature. In addition, the current article also provides the contribution to the literature on self-efficacy and demotivation and resistance to innovation and demotivation. This study provides help to the upcoming literature while examining this topic in the future. This study helps the policymakers to establish the regulations related to the improvement of learning capabilities. This study also guides the relevant authorities while implementing the policies related to attaining sufficient learning capabilities.

This article has several limitations that also the recommendations for the upcoming literature. The current study has taken two independent constructs to predict the insufficient learning capabilities and suggested that future articles should add more factors to predict the insufficient learning capabilities. In addition, the present research has also used the mediating analysis in the framework but ignores the moderating impact and recommended that the upcoming studies add moderating variables in the framework. Moreover, the current study examines China’s second language learning institutions and ignores the other institutions and suggests that future studies should add other institutions to their studies. Finally, the present article has used the questionnaires to collect the data and applied the SPSS-AMOS to analyze the association among variables and ignore the other data collection and analyzing techniques and tools and recommended that future studies incorporate this aspect into their studies.

## Data Availability Statement

The original contributions presented in this study are included in the article/supplementary material, further inquiries can be directed to the corresponding author.

## Author Contributions

YH generated the article idea. MS and YH analyzed the data and wrote the manuscript. TL read and approved the final version. All authors contributed to the article and approved the submitted version.

## Conflict of Interest

The authors declare that the research was conducted in the absence of any commercial or financial relationships that could be construed as a potential conflict of interest.

## Publisher’s Note

All claims expressed in this article are solely those of the authors and do not necessarily represent those of their affiliated organizations, or those of the publisher, the editors and the reviewers. Any product that may be evaluated in this article, or claim that may be made by its manufacturer, is not guaranteed or endorsed by the publisher.
